# Malnutrition awareness and its determinants among Chinese older adults: findings from a cross-sectional survey

**DOI:** 10.3389/fpubh.2025.1638285

**Published:** 2025-08-20

**Authors:** Fangzhou Li, Jingmei Zhang, Difei Wu, Xiyan Yu, Xujiao Chen

**Affiliations:** ^1^Department of Geriatrics, The First Affiliated Hospital of Zhejiang Chinese Medical University (Zhejiang Provincial Hospital of Chinese Medicine), Hangzhou, China; ^2^Department of Emergency, The First Affiliated Hospital of Zhejiang Chinese Medical University (Zhejiang Provincial Hospital of Chinese Medicine), Hangzhou, China

**Keywords:** geriatric assessment, malnutrition, nutrition education, older adults, awareness

## Abstract

**Background:**

Malnutrition is a prevalent but underrecognized health issue among older adults in China. Inadequate awareness may delay detection and intervention, especially in cognitively vulnerable populations. To assess the level of malnutrition awareness and its association with sociodemographic, cognitive, and nutritional factors in a representative sample of older Chinese adults.

**Methods:**

We conducted a cross-sectional survey among 1,227 individuals aged ≥60 years in Zhejiang Province. Malnutrition awareness was measured using a validated 7-item questionnaire. Cognitive status, nutritional risk, frailty, sarcopenia risk, and fall risk were assessed using standard tools. Multivariate logistic regression was used to identify independent predictors of awareness.

**Results:**

Overall, 49.1% of participants demonstrated adequate malnutrition awareness. While most recognized general nutrition concepts (93.5%) and protein supplementation (92.7%), awareness of psychosocial risk factors (e.g., depression) was limited (41.7%). Higher awareness was significantly associated with higher education (OR = 1.38, 95% CI: 1.23–1.55), cohabitation (OR = 1.33, 95% CI: 1.13–1.56), and better cognitive function (*p* < 0.0001). Age, BMI, and frailty were not independent predictors.

**Conclusion:**

Malnutrition awareness among older Chinese adults remains suboptimal, particularly regarding psychological contributors. Targeted education strategies should be stratified by cognitive ability and living context to bridge awareness gaps and support national healthy aging initiatives.

## 1 Introduction

China has entered a stage of deep population aging, with adults aged 65 years and older comprising 15.4% of the total population by the end of 2023 (1). In response, national health policies have increasingly emphasized the development of age-friendly health service systems and support structures (2, 3). Recent government initiatives have launched comprehensive campaigns to promote older adults health, prioritizing domains such as cognitive function, malnutrition, and oral health, which are now recognized as key determinants of functional decline in later life (4–6).

Malnutrition is a common but frequently overlooked condition in the older population, associated with higher risks of frailty, sarcopenia, infection, disability, and mortality (7, 8). In China, the prevalence of malnutrition or nutritional risk ranges from 10% to over 40%, depending on care setting and assessment tool (9, 10). Despite its clinical consequences, public awareness of malnutrition remains underexplored—especially among older adults who are also facing cognitive and sensory decline. Moreover, recent evidence shows that psychosocial factors such as depression, social isolation, and loss of autonomy are often overlooked as contributors to malnutrition, even though they play a central role in geriatric nutrition deterioration (11, 12). For example, the European MAnu study demonstrated that undernutrition in older adults is closely linked with mental health deterioration, yet only a minority of individuals could identify psychological distress as a risk factor (13). In China, while awareness of conditions such as hypertension or diabetes is relatively high among the older adults, awareness of malnutrition remains poorly characterized (14, 15).

Against this backdrop, understanding how older adults perceive the symptoms, causes, and consequences of malnutrition is critical. While most existing research focuses on nutritional screening or intervention outcomes, there is a lack of data on the awareness of malnutrition itself, particularly in relation to cognitive ability, education level, and living situation (16–18). These factors directly affect how older adults access, process, and act on nutrition-related information.

In 2025, China launched the “Weight Management Year” national initiative, reflecting a growing national commitment to promoting healthy aging. This initiative aims to improve population-wide nutritional education, encourage healthy body composition, and prevent chronic diseases, with older adults identified as a priority group. Crucially, the prevention, early recognition, and management of malnutrition among older adults constitute a key element of this broader agenda. However, the effectiveness of such top-down public health strategies depends on a clear understanding of the current level of public awareness regarding malnutrition and its limitations—particularly in vulnerable subgroups (19). Raising awareness about malnutrition—its symptoms, risk factors, and preventive measures—is foundational for the success of national campaigns like “Weight Management Year.” Our study directly supports this national initiative by systematically assessing malnutrition awareness and its associated sociodemographic and health-related determinants in a representative sample of older adults in Zhejiang Province, China. Specifically, we explore how awareness varies according to cognitive function, educational background, living situation, and chronic disease burden. The findings provide actionable evidence to inform targeted, cognition-adapted, and community-based nutrition education strategies, thereby helping to realize the goals of the “Weight Management Year” and China’s broader healthy aging policies.

## 2 Materials and methods

### 2.1 Study design and setting

This was a cross-sectional, questionnaire-based study conducted from communities in Zhejiang, China, who completed questionnaires and somatic function assessments between February 2024 and February 2025. The study aimed to evaluate the awareness of malnutrition and its association with nutritional and health-related parameters among older adults. The Ethics Committee of Zhejiang Provincial Hospital of Chinese Medicine approved this study (Approval No. 2023-KLS-398-02). The clinical trial number is not applicable. Participants who took part in screening for geriatric syndrome were given written informed consent before participating in the study.

### 2.2 Participants

**Figure 1:**
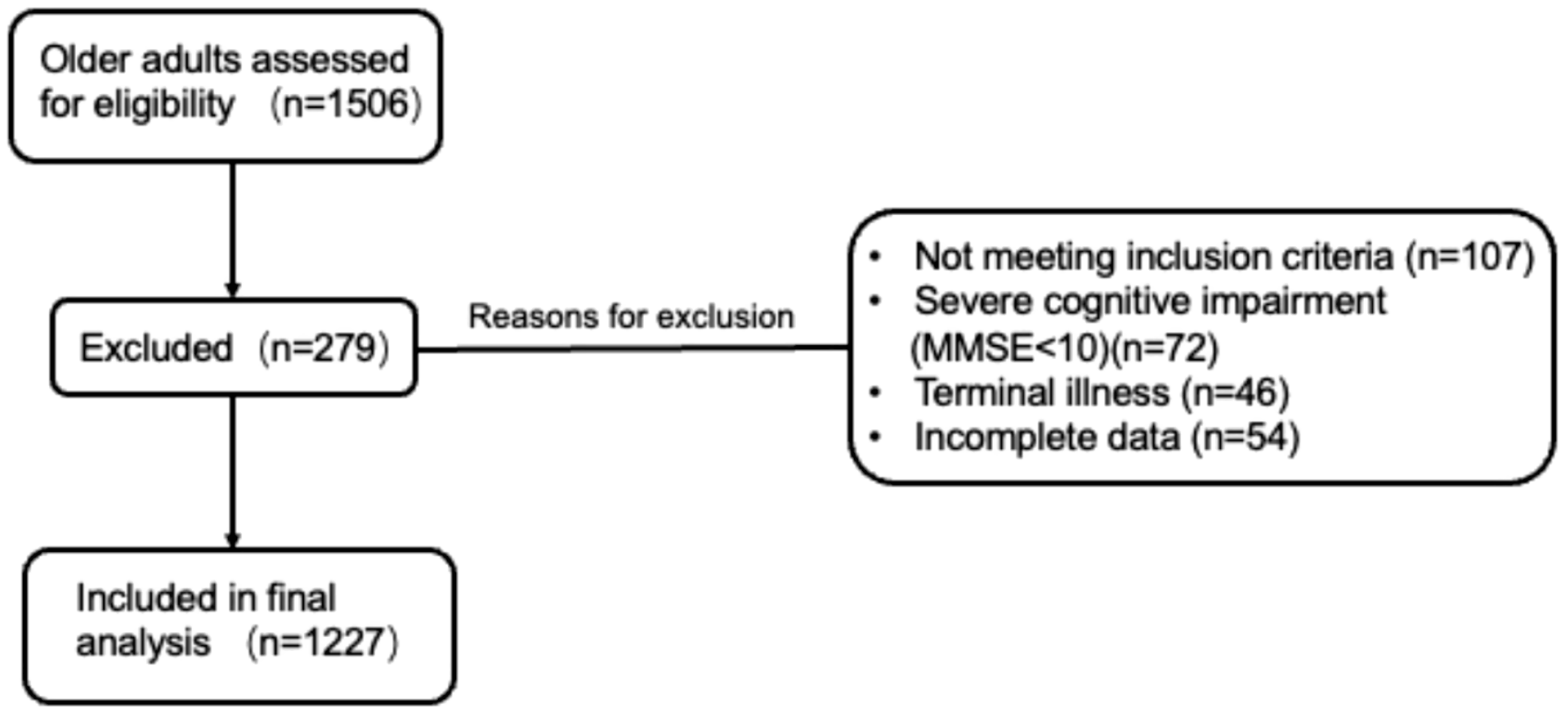
Flowchart.

Participants were consecutively enrolled from both inpatient and outpatient settings of the Department of Geriatrics, Zhejiang Provincial Hospital of Chinese Medicine. The study population thus comprised a mix of older adults with and without chronic diseases, including those attending for routine health screening and comprehensive geriatric assessment. Inclusion criteria were: (1) age 60 or older; (2) ability to provide informed consent and complete the survey independently or with assistance. Exclusion criteria included severe cognitive impairment (MMSE <10), terminal illness (defined as a medical condition with an estimated life expectancy of less than 3 months, including advanced cancer, end-stage organ failure (heart, lung, and kidney), or other severe conditions deemed by clinicians to be beyond active treatment), or incomplete data for key variables. A total of 1,506 older adults were assessed for eligibility. Of these, 279 were excluded for the following reasons: not meeting inclusion criteria (*n* = 107), severe cognitive impairment (MMSE <10; *n* = 72), terminal illness (*n* = 46), or incomplete data (*n* = 54). Finally, 1,227 participants were included in the analysis. The detailed screening and inclusion process is illustrated in Figure 1.

### 2.3 Measurements

Sociodemographic information collected included age, gender, marital status, living arrangement, educational attainment, and employment status. Malnutrition knowledge was assessed using a 7-item questionnaire developed based on our previous work (20), encompassing general nutritional concepts, clinical symptoms, risk factors, and intervention strategies. Consistent with prior methodology, a score of ≥4 correct responses were defined as adequate awareness.

Nutritional Status: Assessed using the Mini Nutritional Assessment–Short Form (MNA-SF) which integrates both subjective and objective indicators, but does not include laboratory or direct muscle strength measurements (21). 12–14: Normal nutritional status; 8–11: At risk of malnutrition; 0–7: Malnutrition.

Cognitive Function: Measured using the Mini-Mental State Examination (MMSE) (22). 27–30: Normal cognitive function; 24–26: Mild cognitive impairment; 18–23: Moderate cognitive impairment; 0–17: Severe cognitive impairment (note: Cut-off values may be adjusted for education level in some studies; in our analysis, MMSE <10 was used as the exclusion criterion for severe impairment).

Frailty: Evaluated by the Fried Frailty Phenotype Scale (23). 0: Robust; 1–2: Prefrail; ≥3: Frail.

Sarcopenia Risk: Assessed by the SARC-F questionnaire (24). 0–3: Low risk of sarcopenia; ≥4: At risk of sarcopenia.

Fall Risk: Estimated with the Morse Fall Risk Scale (25). 0–24: Low risk; 25–44: Moderate risk; ≥45: High risk.

Body Mass Index (BMI) was calculated as weight (kg) divided by height squared (m^2^). <18.5: Underweight; 18.5–23.9: Normal; ≥24: Overweight (per Chinese guidelines).

Data on major chronic diseases (hypertension, diabetes, cardiovascular and cerebrovascular diseases, etc.) were collected for all participants. The presence of these comorbidities was included as an independent variable in multivariate analyses to control for their potential confounding effects on malnutrition awareness and status.

### 2.4 Statistical analysis

All statistical analyses and visualizations were performed using R version 4.2.1 (R Foundation for Statistical Computing, Vienna, Austria), including the use of tidyverse, stats, and ggplot2 packages. Descriptive statistics were presented as means ± standard deviation (SD) for continuous variables and counts (percentages) for categorical variables. Group comparisons between participants who were aware vs. unaware of malnutrition were conducted using: Independent samples t-tests for continuous variables (e.g., MMSE, MNA-SF, and BMI), Chi-square tests for categorical variables (e.g., marital status, education), Multivariate logistic regression was performed with age, cognition (MMSE), BMI, education, living situation, and other significant variables as independent covariates, to control for potential confounding effects, including that of age, to identify independent predictors of malnutrition awareness, with odds ratios (OR) and 95% confidence intervals (CI) reported. Statistical significance was set at *p* < 0.05.

## 3 Results

### 3.1 Participant characteristics

A total of 1,227 older adults were included in the final analysis. The mean age of participants was 73.3 ± 9.8 years, with 54.0% being male. Most participants were married and living with others (73.0%), and the majority were retired. Regarding educational background, a large proportion had received only primary education or were illiterate, whereas a smaller percentage had completed secondary or higher education (Table 1). The mean body mass index (BMI) was 23.0 ± 3.2 kg/m^2^, and the mean Mini-Mental State Examination (MMSE) score was 23.5 ± 5.1. Nutritional assessment indicated a generally good status, with a mean Mini Nutritional Assessment–Short Form (MNA-SF) score of 12.3 ± 2.0. The average Fried frailty score was 1.3 ± 1.4, and the SARC-F score was 1.3 ± 2.2, suggesting low overall prevalence of severe frailty or sarcopenia. The average Morse Fall Risk Score was 30.5 ± 24.0. Multiple chronic diseases were prevalent, with hypertension, diabetes, coronary artery disease, stroke, and osteoporosis being the most frequently reported.

**Table 1:** Demographic characteristics of awareness rate.

Variable	Category	*n* (%)/Mean ± SD
Gender	Male	565 (46.0%)
	Female	662 (54.0%)
Age (years)		73.33 ± 9.80
Marital status	Live together in marriage	896 (73.0%)
	Live apart in marriage	74 (6.0%)
	Widowed	207 (16.9%)
	Divorced	17 (1.4%)
	Unmarried	23 (1.9%)
	Other marital status	10 (0.8%)
Living situation	Live alone	77 (6.3%)
	Live with family	925 (75.4%)
	Nursing home	134 (10.9%)
	With caregiver	17 (1.4%)
	Other situation	74 (6.0%)
Education level	Illiteracy	221 (18.0%)
	Primary school	470 (38.3%)
	Junior school	266 (21.7%)
	High school or three-year college	168 (13.7%)
	University and over	102 (8.3%)
BMI (kg/m^2^)		23.03 ± 3.18
MMSE Score		23.45 ± 5.06
Fried Score		1.29 ± 1.40
SARC-F Score		1.28 ± 2.19
MNA-SF Score		12.26 ± 2.03
MORSE Fall Risk		30.53 ± 24.04

BMI (kg/m^2^): <18.5: Underweight; 18.5–23.9: Normal; ≥24: Overweight (per Chinese guidelines). MMSE: 27–30: Normal cognitive function; 24–26: Mild cognitive impairment; 18–23: Moderate cognitive impairment; 0–17: Severe cognitive impairment. Fried Frailty Phenotype: 0: Robust; 1–2: Prefrail; ≥3: Frail. SARC-F Questionnaire: 0–3: Low risk of sarcopenia; ≥4: At risk of sarcopenia. MNA-SF: 12–14: Normal nutritional status; 8–11: At risk of malnutrition; 0–7: Malnutrition. Morse Fall Risk Scale: 0–24: Low risk; 25–44: Moderate risk; ≥45: High risk.

### 3.2 Awareness of malnutrition

**Figure 2:**
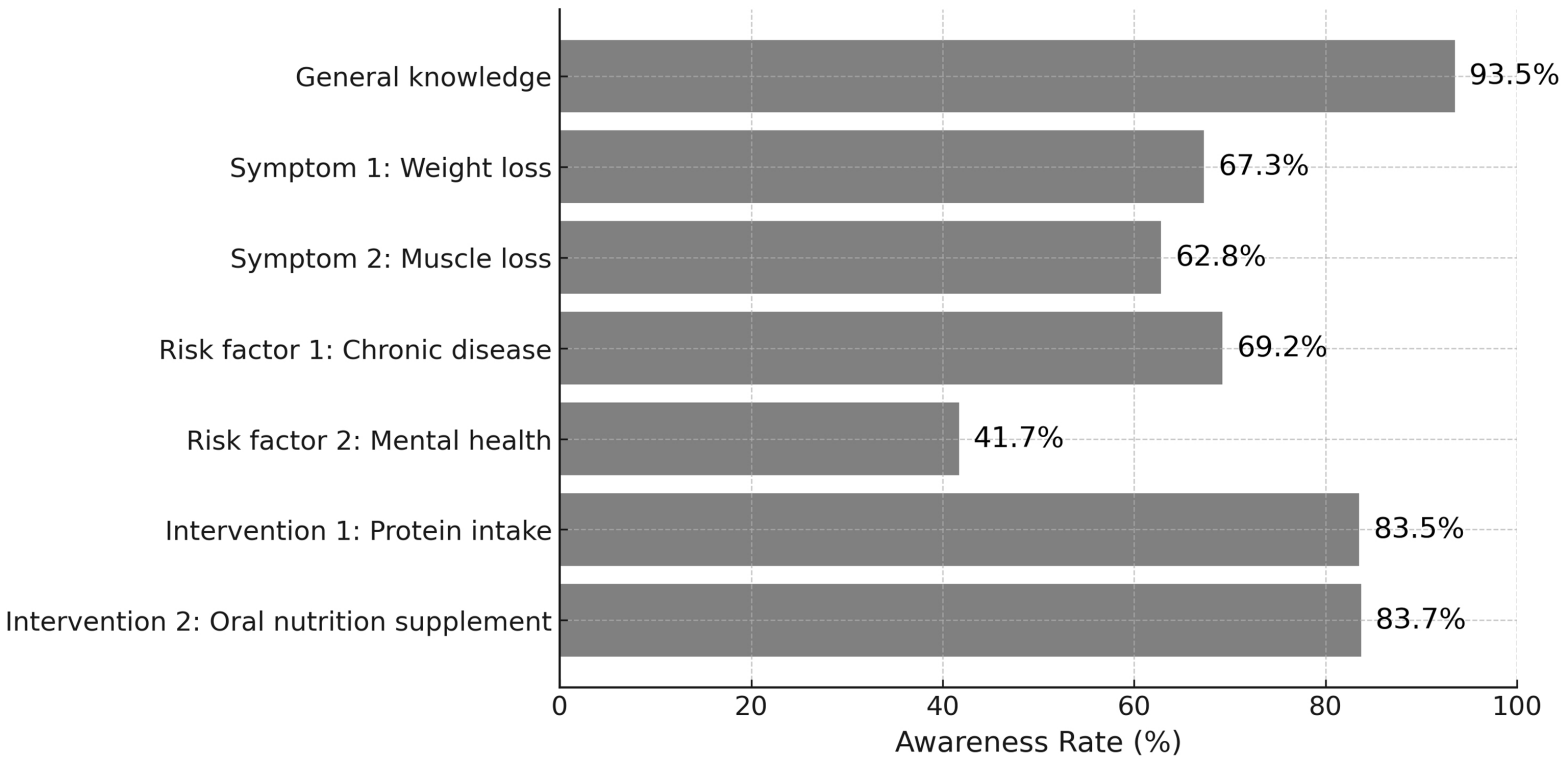
Awareness rate of malnutrition-related items among older adults. Awareness rate of specific malnutrition-related knowledge items among older adults (*n* = 1,227). Items included general understanding, symptom recognition (weight and muscle loss), risk factors (chronic disease, mental health), and recommended interventions (protein intake, oral nutritional supplements). The lowest awareness was seen in psychological risk factors (41.7%), while knowledge of general concept and protein intervention exceeded 90%.

The overall awareness rate of malnutrition was 49.1%, as defined by correctly answering ≥4 out of 7 items in the knowledge assessment scale (Table 2). As shown in Figure 2, the highest awareness was observed for the general concept of malnutrition (93.5%) and recommended interventions such as protein supplementation (92.7%) and use of oral nutrition supplements when dietary intake is inadequate (83.7%). Conversely, awareness was lower for the identification of muscle loss (62.8%) and weight loss (67.3%) as key symptoms, and especially low for recognition of mental health conditions (e.g., depression) as a risk factor (41.7%).

**Table 2:** Awareness of malnutrition by item and overall (*n* = 1,227).

Item	*n* (Aware)	Awareness rate (%)
Malnutrition-1 (general knowledge)	1,147	93.5%
Symptom 1 (weight loss awareness)	826	67.3%
Symptom 2 (muscle loss awareness)	770	62.8%
Risk Factor 1 (chronic disease association)	849	69.2%
Risk Factor 2 (psychological status association)	512	41.7%
Intervention 1 (protein supplementation)	1,138	92.7%
Intervention 2 (use of ONS if diet insufficient)	1,027	83.7%
Overall awareness (≥4 correct answers)	602	49.1%

### 3.3 Univariate analysis of factors associated with awareness

As shown in Table 3, univariate analysis indicated that several sociodemographic variables were significantly associated with malnutrition awareness. Awareness was significantly higher in participants with greater educational attainment (*χ*^2^ = 34.13, *p* < 0.0001) and those living with others (*χ*^2^ = 20.20, *p* = 0.0005). Awareness was also associated with age group (*χ*^2^ = 24.18, *p* < 0.0001) and BMI category (*χ*^2^ = 9.75, *p* = 0.0208). No significant associations were found with gender (*p* = 0.2235), marital status (*p* = 0.2360), or employment status (*p* = 0.6187).

**Table 3:** Univariate analysis of factors associated with malnutrition awareness (*n* = 1,227).

Variable	Chi-square	*p*-value
Gender	1.48	0.2235
Marital status	6.80	0.2360
Living situation	20.20	0.0005
Education level	34.13	<0.0001
Employment status	0.96	0.6187
BMI category	9.75	0.0208
Age group	24.18	<0.0001

### 3.4 Multivariate logistic regression analysis

**Figure 3:**
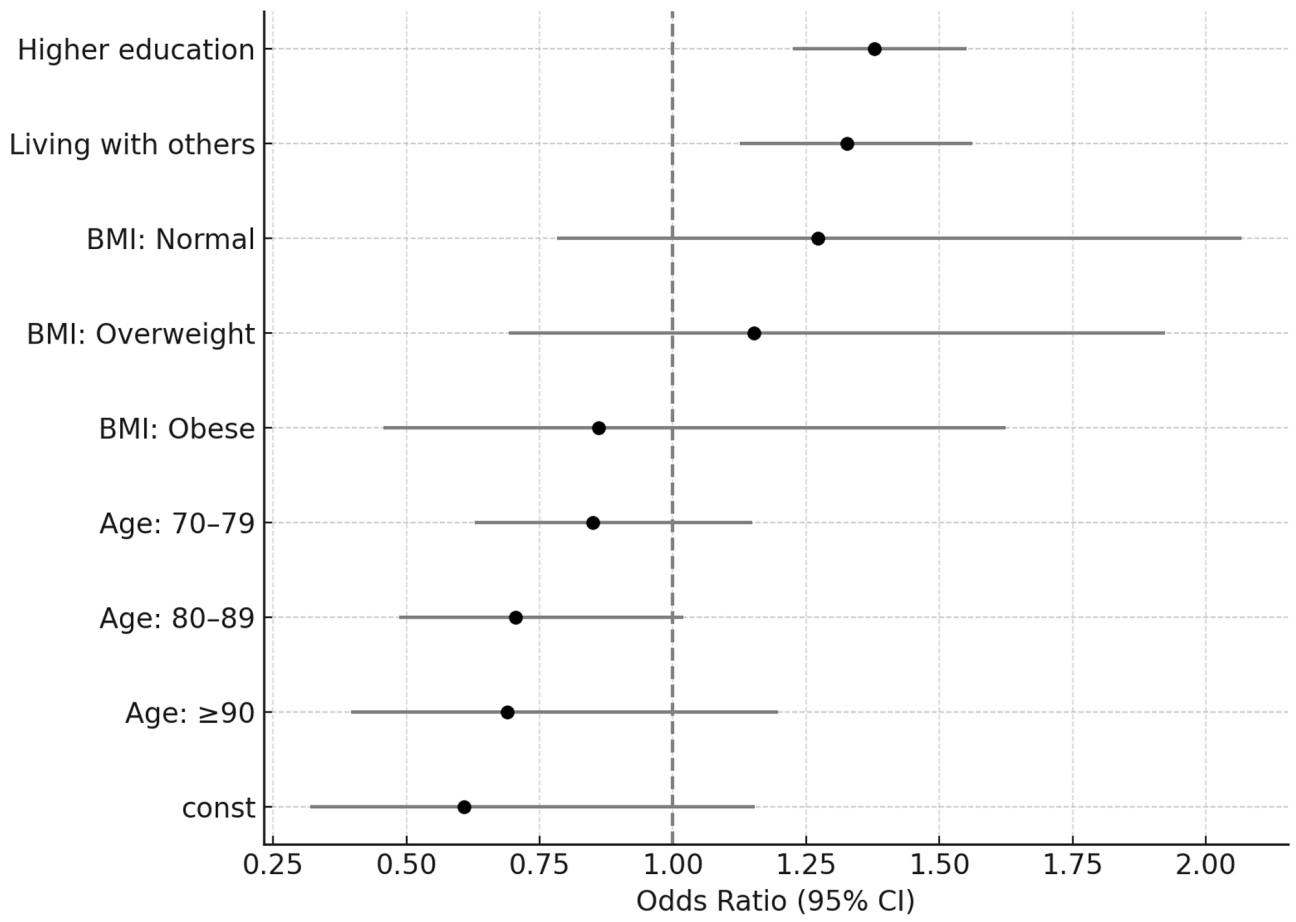
Adjusted odds ratios for malnutrition awareness. Forest plot showing adjusted odds ratios (ORs) and 95% confidence intervals (CI) from multivariate logistic regression for factors associated with malnutrition awareness. Higher educational level and living with others were significantly associated with greater awareness. BMI category and age group were not significantly associated with awareness (*p* > 0.05).

Multivariate logistic regression (Table 4 and Figure 3) identified education level and living situation as independent predictors of malnutrition awareness. Specifically, participants with higher education had significantly greater odds of being aware (OR = 1.38, 95% CI: 1.23–1.55, *p* < 0.0001). Similarly, those living with others had increased odds of awareness compared to those living alone (OR = 1.33, 95% CI: 1.13–1.56, *p* = 0.0008). BMI category and age group, though significant in univariate analyses, were not independently associated after adjustment.

**Table 4:** Multivariate logistic regression analysis of factors associated with malnutrition awareness.

Variable	OR	95% CI	*p*-value
Living situation	1.33	1.13–1.56	0.0008
Education level	1.38	1.23–1.55	<0.0001
BMI category: normal vs. underweight	1.27	0.78–2.07	0.3301
BMI category: overweight vs. underweight	1.15	0.69–1.92	0.5848

### 3.5 Stratified analysis by gender and age

**Figure 4:**
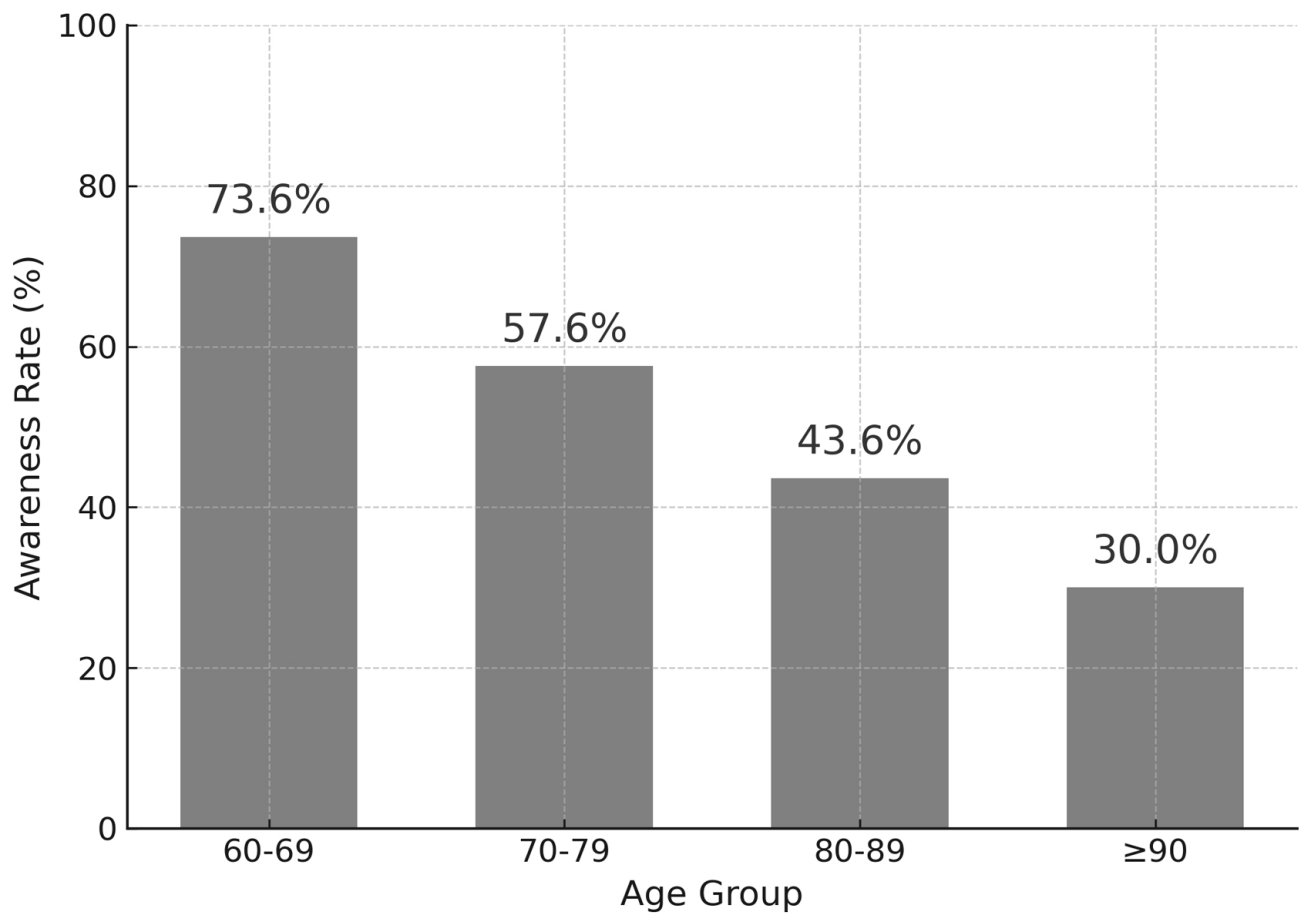
Awareness rate by age group. Awareness rate of malnutrition among older adults by age group (*n* = 1,227). Awareness significantly decreased with increasing age. The highest rate was observed in participants aged 60–69 (73.6%), while the lowest was in those aged 90 and above (30.0%). The trend was statistically significant (*p* < 0.0001).

As shown in Table 5 and Figure 4, stratified analysis by gender revealed no statistically significant differences in malnutrition awareness (male: 54.4%, female: 57.2%, *p* = 0.3289). In contrast, there was a clear and significant inverse trend between age and awareness (*p* < 0.0001). The highest awareness rate was found in participants aged 60–69 years (73.6%), followed by those aged 70–79 (57.6%) and 80–89 (43.6%), with the lowest rate observed among those aged 90 and above (30.0%).

**Table 5:** Stratified analysis of malnutrition awareness by gender and age group.

Stratification	Group	Unaware (n)	Unaware (%)	Aware (*n*)	Aware (%)	*p*-value
Gender	Male	269	45.6%	321	54.4%	0.3289
	Female	198	42.8%	264	57.2%	
Age group	60–69	38	26.4%	106	73.6%	<0.0001
	70–79	161	42.4%	219	57.6%	
	80–89	212	56.4%	164	43.6%	
	≥90	56	70.0%	24	30.0%	

### 3.6 Association between awareness and cognitive status

**Figure 5:**
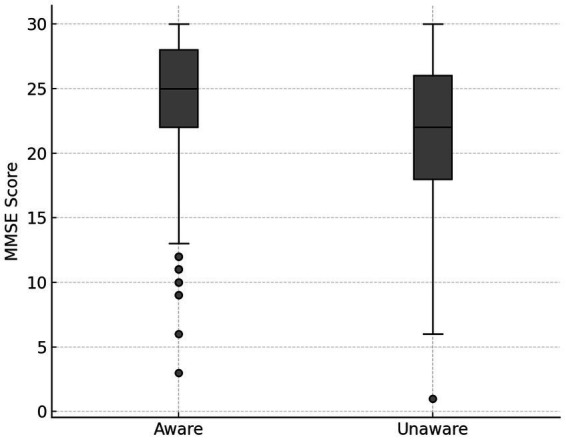
MMSE scores by malnutrition awareness. Comparison of MMSE scores between participants aware and unaware of malnutrition (*n* = 710). Participants with awareness showed significantly higher cognitive function scores (mean MMSE = 24.01 ± 4.59) than those unaware (mean MMSE = 21.05 ± 6.13), *p* < 0.0001.

The association between malnutrition awareness and cognitive function was examined using MMSE scores. As presented in Table 6 and Figure 5, the mean MMSE score was significantly higher in the aware group (24.01 ± 4.59) compared to the unaware group (21.05 ± 6.13, *p* < 0.0001). This suggests that individuals with better cognitive status may have a greater capacity to acquire and retain nutrition-related knowledge.

**Table 6:** Comparison of MMSE scores by malnutrition awareness.

Group	*n*	MMSE score (Mean ± SD)	*p*-value
Aware	574	24.01 ± 4.59	<0.0001
Unaware	136	21.05 ± 6.13	

## 4 Discussion

This study is among the first in China to systematically evaluate malnutrition awareness among older adults and identify its associations with cognitive, educational, and social factors. Our findings reveal that fewer than half (49.1%) of community-dwelling older individuals demonstrated adequate knowledge of malnutrition. While general concepts such as protein supplementation were widely recognized, key clinical symptoms (e.g., unintentional weight loss, muscle wasting) and psychosocial contributors (e.g., depression) were often overlooked. These results underscore a critical knowledge gap that may compromise early recognition and management of malnutrition in aging populations.

### 4.1 International comparison and psychosocial dimensions

Our results are consistent with previous international and Chinese studies that suggest awareness of malnutrition is low among older adults. A nationwide survey in Germany revealed that fewer than half of older adults participants could identify basic signs of malnutrition, such as unintended weight loss or appetite reduction (26). Similarly, a systematic review covering low- and middle-income countries highlighted that most older adults lack accurate knowledge regarding malnutrition symptoms, risk conditions, and treatment options (27). In China, although awareness of chronic diseases like hypertension is high, malnutrition remains a neglected issue in public health education (14, 15).

Particularly noteworthy is the low awareness of psychological contributors to malnutrition. In our study, only 41.7% of participants recognized mental health as a relevant risk factor. This is concerning given robust evidence that depression, loneliness, and cognitive impairment are strongly linked to nutritional decline in older populations (11, 12). The observed cognitive-nutritional interplay emphasizes the need for integrated health education addressing both physical and psychosocial dimensions.

### 4.2 Cognitive and social determinants of nutritional literacy

Our multivariate model confirmed that higher educational attainment, intact cognitive function, and cohabitation with others were significant independent predictors of malnutrition awareness. These results align with previous research indicating that educational attainment enhances health literacy and access to reliable nutrition information (28). Similarly, living with others may increase exposure to health messages and social encouragement for healthy behaviors (29). Our findings support the call for socially contextualized interventions targeting isolated or less-educated older adults (30, 31). While age group and BMI category were significant in univariate models, they did not remain independent predictors after adjustment. This may reflect underlying confounding with cognitive function and education level, which were not evenly distributed across age strata.

We found a statistically significant association between cognitive status and malnutrition awareness, with higher MMSE scores observed in the aware group. This finding is in line with previous research and can be explained by several interrelated mechanisms (32, 33). First, individuals with better cognitive function generally possess higher health literacy, allowing them to more effectively process, understand, and retain health information, including knowledge about nutrition and malnutrition prevention. Second, intact cognitive abilities—such as memory, attention, and executive function—facilitate planning, decision-making, and self-management, enabling older adults to recognize symptoms, understand risk factors, and act on nutrition-related guidance. Furthermore, cognitively healthy older adults are more likely to participate in community and social activities, increasing their exposure to health education and peer support. Conversely, cognitive decline impairs these capacities, reducing one’s ability to acquire and recall health knowledge, recognize early signs of malnutrition, and adhere to dietary recommendations (34). In addition, memory loss, impaired judgment, and eating difficulties may directly contribute to undernutrition. These findings underscore the need to tailor educational strategies to the cognitive abilities of older adults. Public health interventions should consider simplified, visual, or repetitive messaging and actively involve caregivers or family members for those with cognitive impairment. Addressing these cognitive barriers is essential for the effective delivery of nutrition education and for maximizing the reach and impact of healthy aging policies at the population level. Evidence suggests that deficiencies in specific micronutrients, particularly vitamin D, may negatively impact cognitive function, which in turn may increase the risk of malnutrition among older adults. While our study did not assess vitamin D or other micronutrient status, the potential role of such deficiencies in the cognitive-nutritional pathway warrants further investigation. Future research should incorporate objective measures of key micronutrients (such as vitamin D) to better understand their mediating effects between cognition and nutrition, and to inform more targeted intervention strategies.

### 4.3 Policy implications: toward cognition-stratified nutrition education

China’s 2025 “Weight Management Year” initiative provides a timely national framework for addressing malnutrition. However, our findings indicate that universal nutrition messaging may fail to reach subgroups with cognitive or educational vulnerabilities. To maximize impact, we propose a cognition-stratified nutrition education model guided by the following principles:

1. Cognitive tiering: Education delivery formats (e.g., digital modules, pictorial handbooks, and group sessions) should be matched to cognitive ability and literacy level. For cognitively intact individuals, app-based self-learning tools may be appropriate, while those with mild impairment may benefit from simplified visuals or family-assisted sessions. Those with significant decline may require direct caregiver-targeted education.
2. Social integration: Family caregivers and community health workers should be mobilized as co-educators, particularly for isolated or homebound older adults. Education can be embedded into existing community outreach programs or long-term care assessments.
3. Mental health inclusion: Nutrition education content should explicitly address emotional health, appetite suppression from depression, and socially embedded risks. This would align with current evidence that psychological distress is a major, yet underrecognized, contributor to undernutrition.
4. Monitoring and feedback: We recommend incorporating a brief, validated nutrition knowledge screening tool—such as the 7-item scale used in this study—into routine geriatric health evaluations. This would allow public health programs to track awareness trends and evaluate the effectiveness of interventions over time.

Furthermore, we advocate the creation of a risk stratification framework to guide prioritization of interventions. Our results suggest that individuals with (1) MMSE score <22, (2) educational attainment below primary level, and (3) living alone status may constitute a high-risk triad for poor nutrition literacy. These criteria can be operationalized into community-level screening algorithms to triage education efforts efficiently.

### 4.4 Relevance to older adults health system reform

This study supports the national agenda of building elder-friendly health systems by offering data-driven, tiered intervention strategies that reflect real-world heterogeneity in older adults’ cognitive and social functioning. Our findings emphasize that knowledge inequities are not merely informational but structurally rooted in cognitive capacity, education, and living arrangements.

From a health systems perspective, we recommend the integration of nutrition knowledge assessment into standardized geriatric screening protocols. In community health centers, pairing tools such as the MNA-SF with a brief cognitive screen (e.g., MMSE or MoCA) and a simplified malnutrition knowledge test could enhance early detection of “hidden risk” individuals—those who are not yet malnourished but lack the awareness needed to prevent it.

Moreover, public health authorities could consider embedding nutrition knowledge indicators into national aging surveillance systems, particularly within programs like the Healthy Aging Strategy or regional electronic health record platforms. This would enable systematic tracking of population-level awareness gaps and guide longitudinal policy adjustments.

To ensure long-term sustainability, we also advocate investment in neuro-adaptive education technologies, such as touchscreen kiosks in community centers with animated guidance, or voice-assisted modules for visually or cognitively impaired individuals. These tools should be designed in alignment with behavior change theories, using reinforcement and repetition to optimize retention. By transitioning from a reactive to a proactive prevention model, and by embedding educational sensitivity into public health architecture, China’s response to malnutrition in aging societies can become both more precise and equitable.

### 4.5 Strengths and limitations

This study’s strengths include its large representative sample, use of validated tools (e.g., MMSE, MNA-SF, SARC-F), and integration of multidimensional geriatric health indicators. Nevertheless, several limitations should be acknowledged. Due to the cross-sectional nature of our study, we cannot infer causality or the directionality of the relationship between cognitive decline and malnutrition. Evidence suggests a bidirectional association: cognitive impairment may contribute to nutritional decline, while poor nutritional status may in turn accelerate cognitive deterioration. Future prospective and interventional studies are needed to elucidate these mechanisms and inform targeted prevention strategies. As all subjects were recruited from hospital-based services, this may limit the generalizability of findings to the broader healthy community-dwelling older adults population. And In our study, 78.8% of participants were outpatients and 21.2% were inpatients. While including both groups helps capture a wider range of older adults, these differences may introduce selection bias. For instance, inpatients often have lower functional status and greater health needs, which could influence their awareness of malnutrition. Future research should use stratified analyses or population-based samples to better address these potential biases. Lastly, in China, rapid socioeconomic changes have created substantial generational, cultural, and regional differences in education, nutrition knowledge, dietary habits, and access to food among older adults. Malnutrition awareness may therefore vary widely, especially between urban and rural areas. Although we adjusted for education and some socioeconomic factors, residual confounding from unmeasured variables such as dietary culture and region may remain. As our study mainly involved urban and peri-urban participants, with few from rural areas, future research should include more diverse samples and detailed dietary and socioeconomic data to better assess these generational and regional differences in malnutrition awareness.

## 5 Conclusion

This study highlights that malnutrition awareness among older adults in China is insufficient, especially regarding clinical symptoms and psychosocial risk factors. While general nutrition knowledge is relatively widespread, deficits remain in recognizing early warning signs of undernutrition. Cognitive function, education level, and cohabitation status emerged as key determinants of awareness, indicating the need for stratified, cognition-sensitive education strategies. Standardized health messaging alone may not effectively reach vulnerable subgroups, such as those with cognitive decline or low literacy. Future research should move beyond cross-sectional associations to rigorously evaluate, through longitudinal and interventional studies, whether improving malnutrition awareness among older adults leads to measurable improvements in clinical outcomes, such as nutritional status, functional ability, quality of life, and morbidity. Moreover, it is crucial to test the effectiveness and scalability of cognition-stratified nutrition education models in diverse real-world settings—including urban, rural, and minority communities—to provide robust, evidence-based recommendations for national and regional health policies. Only through such translational research can we bridge the gap from descriptive findings to actionable public health strategies that promote healthy aging at scale.

## Data Availability

The raw data supporting the conclusions of this article will be made available by the authors, without undue reservation.
